# Pine Shoot Blight Driven Seasonal Variations in Fungal Assembly of *Pinus elliottii* Rhizosphere

**DOI:** 10.3390/microorganisms13112476

**Published:** 2025-10-29

**Authors:** Xiang Duan, Wenhao Li, Jiechen Zhou, Xingzhou Chen, Pingan Chen, Guoying Zhou

**Affiliations:** 1 College of Forestry, Central South University of Forestry and Technology, Changsha 410004, China; 2 Key Laboratory of National Forestry and Grassland Administration on Control of Artificial Forest Diseases and Pests in South China, Central South University of Forestry and Technology, Changsha 410004, China; 3 Xiangtan Forestry Research Institute of Science, Xiangtan 411100, China

**Keywords:** pine shoot blight, microbial functional diversity, environmental factor, seasonal variation

## Abstract

Ectomycorrhizal fungi (ECMF) function as critical mediators connecting plant roots and associated microorganisms. These fungi establish intimate associations with the root systems of diverse higher plants, particularly Pinaceae species, constituting essential components of forest ecosystems. The current understanding of ECMF community structure in *Pinus elliottii* and its potential associations with soil characteristics remains inadequate. This investigation examined seasonal variations in rhizosphere soil physicochemical properties and fungal community dynamics between susceptible (YB) and healthy (YJ) *P*. *elliottii* using amplicon sequencing. The results demonstrated significant seasonal differences in fungal community composition between YB and YJ. Dominant ECMF genera exhibited distinct distribution patterns, with *Rhizopogon* predominating in YJ and *Tricholoma* in YB. Correlation analyses revealed strong associations between these ECMF taxa and key soil parameters (available potassium, total phosphorus, and available phosphorus), indicating substantial seasonal influences of phosphorus and potassium cycling on ECMF development. Ericoid mycorrhizal fungi displayed higher abundance in YJ samples during spring, suggesting their dual role in facilitating nutrient acquisition and enhancing host plant resilience against biotic and abiotic stresses. These findings provide novel insights into seasonal dynamics of fungal communities in *P*. *elliottii* ecosystems and offer practical implications for sustainable plantation management under global change scenarios.

## 1. Introduction

Mycorrhiza represents a mutualistic symbiosis between specific soil fungi and plant roots [1]. Fungal hyphae absorb organic compounds like sugars from host plants to sustain growth [2,3]. In return, fungi supply nutrients and water to hosts, enhancing plant growth and improving adaptation to extreme environmental stressors [4]. There are four main types of mycorrhizas based on the criteria of morphological differentiation of root tissues and host plant lineages: arbuscular mycorrhizas (AMs), ectomycorrhizas (EcMs), ericoid mycorrhiza (ErM) and orchid mycorrhizas (OrMs) [5]. Arbuscular mycorrhizas (AMs) are defined by the presence of arbuscules that normally form in root cortex cells [6]. Ericoid mycorrhizas (ErMs) are limited to members of Ericaceae, excluding some subfamilies (Monotropoideae, Arbutoideae and Enkianthoideae), but including the Diapensiaceae. Orchid mycorrhizas (OrMs) are confined to the Orchidaceae [5]. Ectomycorrhizal fungi (ECMF) serve as key mediators connecting root systems, symbiotic partners, and associated microorganisms [7,8]. These fungi form close associations with diverse higher plants, such as Pinaceae species, and constitute essential components of forest ecosystems [9,10]. ECMF improve soil quality and mitigate environmental stresses including drought, pathogens, and heavy metals [11,12]. Their functions are vital for nutrient cycling, biodiversity maintenance, and ecosystem stability in forests [13,14,15]. ECMF communities exhibit spatiotemporal heterogeneity influenced by environmental conditions and host interactions [16,17,18]. Understanding ECMF diversity and spatial dynamics is crucial for tree health and productivity. ECMF further represent major soil microbial groups that colonize fine root tips of plants [19].

The abundance of microbial communities exhibits distinct seasonal patterns, with their influence surpassing that of nutrients and other disturbances [20,21]. Seasonal changes can influence fungal communities by altering climatic factors and the supply of carbon and phosphorus in host plants. For example, when plants are growing vigorously, they require increased phosphorus supply, which may favor the adaptation of fungal communities capable of providing substantial phosphorus to their hosts [22]. When plants flower or age during the year, their nutrient requirements are expected to decrease, and the transfer of carbon to fungal symbionts will correspondingly diminish [23]. Changes in resource allocation alter the competitive capacity of plant–fungal symbiotic associations, implying that seasonal shifts in fungal community assembly may vary not only with seasons and plant life stages but also with differences in plant–fungal interactions.

Pine shoot blight represents one of the most widespread forest diseases in China. It was first identified in *P. massoniana* and *P. elliottii* forests in Hunan Province in 1976. Subsequent discoveries occurred in other Chinese regions. This disease has caused severe damage to pine forests in many areas or countries. China officially recognized it as a key forest disease for scientific research in 1999. *Sphaeropsis sapinea*, a fungus within the *Sphaeropsidales* order of *Deuteromycotina*, causes pine shoot blight. This pathogen exhibits host dependency. Infection typically occurs under stressful environmental conditions when host plant vigor declines. Infected hosts display distinctive symptoms. These include progressive dieback of needles, terminal buds, new shoots, and fascicles. Additional symptoms encompass stem cankers, resin bleeding, browning, drying, and root rot. Pine shoot blight affects numerous coniferous genera. These genera include *Pinus*, *Abies*, *Larix*, *Thuja*, and *Cedrus*. Primary hosts notably include *P. massoniana*, *P. elliottii*, *P. taeda*, and *P. radiata* [24,25,26].

Pedersen demonstrated enhanced phosphorus uptake in *P. elliottii* seedlings following inoculation with *Pisolithus* species [27]. Chen reported that inoculating *P. elliottii* and other pines with ECMF can control forest diseases [28]. Wang inoculated *P. massoniana* seedlings with multiple ECMF species [29]. Subsequent measurements of growth, physiology, and nutrient absorption identified *Suillus luteus*, *Cantharellus cibarius*, *Pisolithus tinctorius*, and *Cenococcum geophilum* as high-performing species. Studies also detail the mycorrhiza formation process between *Suillus grevillea* and *P. massoniana*. This process involves distinct pre-symbiotic and symbiotic stages. During development, the Hartig net forms before the mantle [30]. ECMF not only enhances disease resistance by influencing host plants’ nutrient uptake efficiency from different fertilizers, but also acts as a mechanical barrier by competing with pathogens for nutrients or inducing morphological changes in plant root structures [31,32]. Inducing systemic defense responses in host plants is also a common mechanism by which ECMF enhances plant disease resistance. For example, it achieves this by boosting antioxidant enzyme activity within the plant, promoting seedling growth, and thereby suppressing disease occurrence [33]. Additionally, ECMF itself can synthesize and secrete secondary metabolites such as defensive enzymes, alkaloids, and phenolic compounds to suppress the development of host pests and diseases [34].

Research on the physiology of ECMF in *P. elliottii* is established. However, investigations into their community structure remain limited. This study examined susceptible and healthy *P. elliottii* across seasons. High-throughput sequencing analyzed fungal community dynamics and the influence of soil factors. This approach aims to provide a theoretical foundation and technical support for the sustainable management of future *P. elliottii* plantations.

## 2. Materials and Methods

### 2.1. Sampling Site

The study area is situated within the Yunyang Mountain State-Owned Forest Farm, Chaling County, Zhuzhou City, Hunan Province (113°30′37.88″ E to 113°27′28.43″ E, 26°48′3.10″ N to 26°43′26.39″ N). This region borders both Hunan and Jiangxi provinces, encompassing a total area of 8688.70 hectares. Characterized by a humid subtropical climate, the area experiences a mean annual temperature of 14.5 °C and receives an average annual precipitation of 1658 mm. Extensive plantations of *P. elliottii* within the site provide favorable conditions for spatial comparability studies.

### 2.2. Sample Settings and Soil Sampling

After investigation, it was found that there are large areas of *P. elliottii* infected with pine shoot blight in the sampling site. This study set up a 30 × 30 m plot in both diseased and healthy areas, with each plot containing 30 plants. We randomly selected target trees with uniform growth and spacing ≥ 3 m between them. Root samples (15 cm in length) were collected from the 10 cm soil layer near the tree base using the “root-tracing method” [35]. Rhizosphere soil adhering to fine roots after gentle shaking was brushed into sterile bags using a sterile brush. After removing debris (e.g., residual roots, leaf litter, stones), the samples were homogenized and stored in dry ice-cooled containers. They were transported to the laboratory for preservation at −80 °C prior to high-throughput sequencing [36]. Additional subsamples were air-dried for physicochemical analysis. In April and October of the same year, rhizosphere soil was collected from diseased and healthy plants. In addition, to minimize interference, each sampling was conducted within a week without any special weather changes. The experiment comprised four treatments with four replicates each, totaling 16 sequencing samples.

### 2.3. Testing of Soil Physicochemical Properties

Soil pH was determined using an acidity meter (1:2.5). Determination of soil organic carbon (SOC) was performed by external heating of potassium dichromate. Total phosphorus (TP) determination of molybdenum antimony was carried out using colorimetry. Determination of available phosphorus (AP) was performed using the sodium hydrogen carbonate solution/Mo-Sb anti-spectrophotometric method. Rapid available potassium (AK) was determined with flame spectrophotometry. Determination of total potassium (TK) was carried out using a flame photometer. The content of alkali-hydrolyzed nitrogen (AHN) was determined with the alkali-diffusion method [37].

### 2.4. DNA Extraction and Sequencing of PCR Amplicons

The collected soil samples were sent to Shanghai Majorbio Bio-pharm Technology Co., Ltd. for absolute quantitative sequencing of fungal diversity targeting the ITS region. Total microbial DNA was extracted using the E.Z.N.A.^®^ Soil DNA Kit (Omega Bio-tek, Norcross, GA, USA) following the manufacturer’s protocol. The quality of extracted DNA was assessed with 1% agarose gel electrophoresis, while concentration and purity were determined using a NanoDrop 2000 spectrophotometer (Thermo Scientific, Waltham, MA, USA). Fungal amplicon libraries were generated by amplifying the ITS1 region, and the purified DNA samples were stored at −80 °C for subsequent analysis [38,39].

The pooled DNA served as the template for PCR amplification of the ITS1 region using barcode-tagged forward primer ITS1F (5′-CTTGGTCATTTAGAGGAAGTAA-3′) and reverse primer ITS2 (5′-GCTGCGTTCTTCATCGATGC-3′). The PCR conditions followed those described by Wang Yonglong [40]. Amplification products were purified using a DNA gel extraction kit (PCR Clean-Up Kit, Yuhua, China) and quantified with a Qubit 4.0 fluorometer (Thermo Fisher Scientific, MA, USA). Final sequencing was performed on the Illumina NextSeq 2000 platform.

### 2.5. Statistical Analyses

We used software fastp (https://github.com/OpenGene/fastp, version 0.20.0) (Accessed: 6 July 2025) to control the original sequencing sequence [41,42], and the software FLASH (https://ccb.jhu.edu/software/FLASH/index.shtml, version 1.2.7) (Accessed: 6 July 2025) for splicing [43]. We used the software UPARSE based on 97% similarity (http://drive5.com/uparse/, version 7.1) (Accessed: 6 July 2025). Operation of a sequence taxon (operational taxonomic units, OTUs) was performed for clustering and culling of chimeras [44,45]. Using an RDP classifier (version 2.2) (Accessed: 6 July 2025), we made a species classification note for each sequence, setting the contrast threshold of 70% compared to unite9.0/its_fungi [46].

High-throughput sequencing data analysis was performed on the Majorbio Cloud Platform (https://cloud.majorbio.com/) (Accessed: 6 July 2025). Alpha diversity indices (Shannon, Simpson, Coverage, and Chao1) were calculated using Mothur. Community composition bar and pie charts were generated with R software (version 3.3.1). Nonmetric multidimensional scaling (NMDS) analysis based on Bray–Curtis distance was conducted using the Vegan package in R, followed by redundancy analysis (RDA). Correlation analysis between microbial abundance and soil properties was performed using IBM SPSS Statistics 23. Fungal functional (http://www.funguild.org/) classification was accomplished via FUNGuild. Species co-occurrence networks were constructed using Networkx (version 1.11), followed by single-factor correlation network analysis. The top 20 most abundant features were selected to calculate correlation coefficients, revealing significant Pearson correlations (*p* < 0.05). One-way ANOVA was used to analyze the differences between treatment groups, and Tukey’s test was used to separate the means.

## 3. Results

### 3.1. Soil Physicochemical Analyses

Significant differences in pH were observed between YJ and YB across seasons, with higher values recorded in spring than autumn. SOC, AHN, AP, TP, and TK contents were consistently greater in YB than YJ throughout both seasons. In contrast, AK exhibited an opposite trend (YJ > YB). Seasonal variations revealed increasing trends for TP and TK (October > April), whereas AHN and AK showed decreasing patterns (April > October) (Table 1).

### 3.2. Community Diversity Analysis

Alpha diversity analysis was performed on the ITS rDNA diversity indices of YB and YJ across different seasons. As shown in Table 2, the community coverage of all samples exceeded 99%, indicating that the experimental sequencing results could accurately reflect the actual situation of the tested soil samples. The Shannon index of the spring soil samples was significantly higher than that of the autumn soil samples, and the fungal community diversity of YB was lower than that of YJ. For the Simpson index, there was no significant difference between YB and YJ, while the index of the autumn soil samples was slightly higher than that of the spring soil samples. In terms of species richness, the Chao index of the spring soil samples was higher than that of the autumn soil samples, reaching a significant level. This suggests that the fungal community diversity of the spring soil samples is greater than that of the autumn soil samples.

### 3.3. Analysis of Fungal Community Structure and Composition

**Figure 1:**
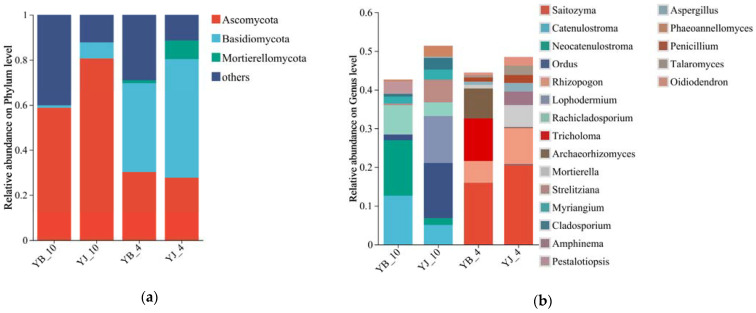
Composition of rhizosphere soil fungal communities. (**a**) Relative abundance at the phylum level; (**b**) relative abundance at the genus level (top 20). The abundance of fungal species is represented by the height of the columns.

The rhizosphere fungi were classified into 11 phyla, 49 classes, 125 orders, 272 families, and 456 genera (Supplementary Table S1). Seasonal analysis of YB and YJ sites revealed *Ascomycota* and *Basidiomycota* as the dominant phyla in the *P*. *elliottii* rhizosphere fungal communities (Figure 1a). The relative abundance of these two dominant phyla varied across seasons, with *Basidiomycota* predominating in the spring soil samples and *Ascomycota* showing higher abundance in the autumn samples. At site YB-10, the predominant fungal genera were *Catenulostroma*, *Rachicladosporium*, and *Neocatenulostroma*, while YJ-10 was dominated by *Ordus*, *Catenulostroma*, and *Lophodermium*. The YB-4 site exhibited *Saitozyma*, *Archaeorhizomyces*, and *Tricholoma* as the dominant genera, whereas YJ-4 was characterized by *Saitozyma*, *Mortierella*, and *Rhizopogon* (Figure 1b). Notably, the compositional structure of fungal communities differed markedly between YJ and YB sites across seasons.

**Figure 2:**
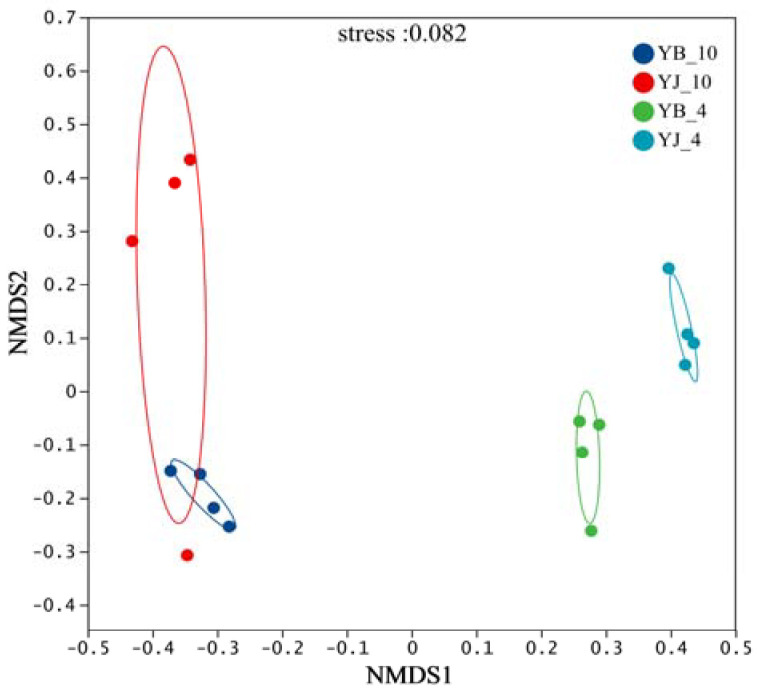
Nonmetric multidimensional scaling (NMDS) analysis of soil fungal community. Each spot represents a sample, and the circles contain samples with a 90% confidence interval for the groups.

Nonmetric multidimensional scaling (NMDS) simplifies complex ecological patterns by extracting principal components that reflect differences among samples from high-dimensional data. Based on the Bray–Curtis distance matrix at the species level, the NMDS analysis revealed clear distinctions between samples (Figure 2). The stress value (0.0016, <0.2) confirmed the reliability of the ordination. The samples from different seasons were largely separated, while those from the same season exhibited greater similarity in fungal community structure. These findings indicate variations in rhizosphere soil fungal composition across seasons and tree health conditions [47].

### 3.4. Effects of Environmental Factors on Fungal Community Structure

**Figure 3:**
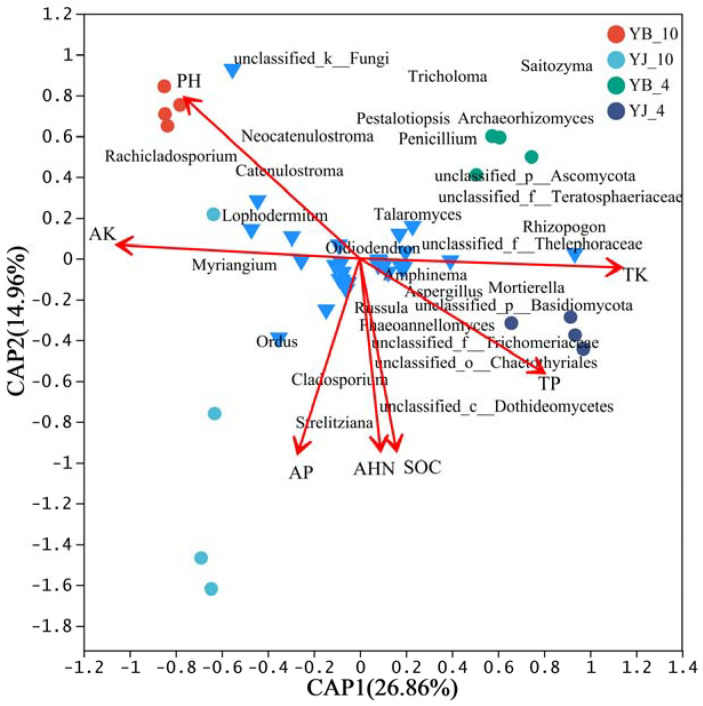
Redundancy analysis (RDA) of environmental factors and fungal communities. Red arrows represent the weights of corresponding variables. Longer arrows indicate stronger explanatory power and greater influence of the variable on the RDA space, while shorter arrows indicate weaker explanatory power. The angle between two arrows reflects their correlation. An angle of 0° indicates perfect positive correlation, an angle of 90° indicates no correlation, and an angle of 180° indicates perfect negative correlation.

Redundancy analysis (RDA) was performed on 30 fungal genera, showing significant differences between YB and YJ in spring and autumn, along with soil environmental factors (pH, SOC, AHN, AP, TP, AK, and TK). The quadrant distribution, arrow length, and angles of fungal taxa and environmental factors were used to assess their correlations with seasonal microbial community structure. As shown in Figure 3, the selected environmental factors explained 41.82% of the variation in fungal community composition across seasons. RDA1 and RDA2 accounted for 26.86% and 14.96%. The primary environmental factors correlated with the fungal community structure in YJ-10 were, in descending order of significance, AP, pH, and AK. For YJ-4, TK, TP, AHN, and SOC showed correlations. Additionally, dominant differentially abundant fungal genera displayed distinct environmental preferences: *Catenulostroma*, *Neocatenulostroma*, and *Rachicladosporium* were linked to pH and AK; *Strelitziana* was linked to AP; *Mortierella* was linked to TK, TP, AHN, and SOC.

**Figure 4:**
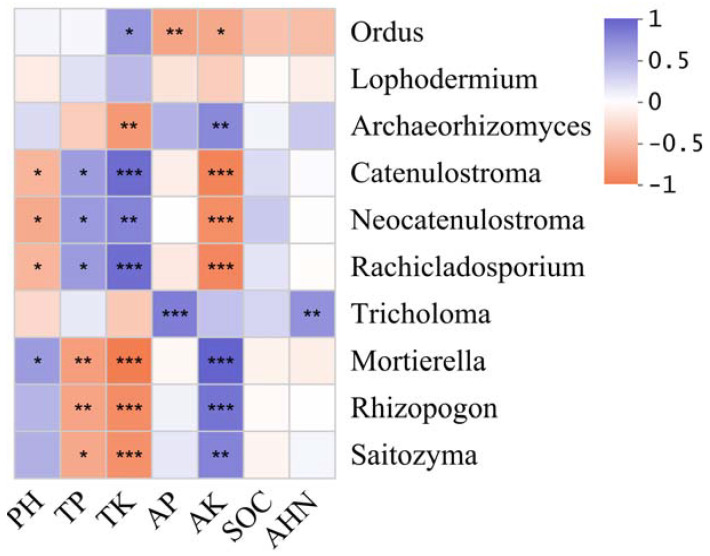
Correlation heatmap between fungal and environmental factors. The blue and red boxes represent positive and negative correlations, respectively. * *p* < 0.05; ** *p* < 0.01; *** *p* < 0.001.

To elucidate the influence of environmental factors on fungal community composition, key fungal genera exhibiting strong environmental correlations were identified (Figure 4). The analysis revealed that TP showed highly significant negative correlations with *Rhizopogon* and *Mortierella*. Similarly, AK demonstrated highly significant negative correlations with *Rachicladosporium*, *Neocatenulostroma*, and *Catenulostroma*, but exhibited highly significant positive correlations with *Rhizopogon*, *Archaeorhizomyces*, *Mortierella*, and *Saitozyma*. In contrast, TK displayed opposite correlation patterns with dominant fungal genera compared to AK. Additionally, AP and AHN were both positively correlated with *Tricholoma* at a highly significant level.

### 3.5. Fungal Functional Prediction Analysis

**Figure 5:**
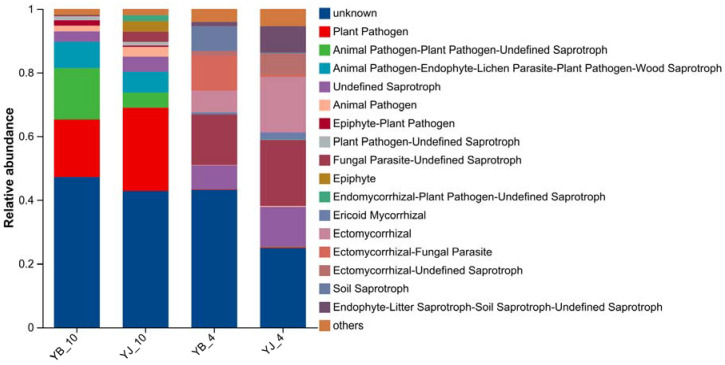
Functional prediction of rhizosphere fungal communities in healthy vs. diseased soils. The abundance of fungal functional guilds is represented by the height of the columns.

Fungal functional guilds in YB and YJ were predicted using the FUNGuild database. As illustrated in Figure 5, seven trophic modes were identified: pathotroph/saprotroph/symbiotroph (14.17%), pathotroph (12.78%), saprotroph (10.02%), symbiotroph (7.56%), pathotroph/saprotroph (6.26%), saprotroph/symbiotroph (4.91%), and pathotroph/symbiotroph (4.32%). The predominant trophic type was the tripartite pathotroph/saprotroph/symbiotroph guild, followed by specialized pathotrophic and saprotrophic guilds.

Saprotrophic fungi derive nutrients from organic matter decomposition, converting plant residues and animal waste into mineral nutrients available for plant uptake. Our results revealed pronounced differences in trophic guild composition between YB and YJ, with seasonal variations. Notably, functional fungal communities (including plant pathogens, animal pathogen/plant pathogen/undefined saprotrophs, and animal pathogen/endophyte/lichen parasite/plant pathogen/wood saprotrophs) increased substantially from spring to autumn. Spring samples exhibited ericoid mycorrhizal colonization, which enhances host plant growth, nutrient acquisition (particularly nitrate and organic nitrogen assimilation), and stress resistance [48]. The higher abundance of ericoid mycorrhizae in YJ compared to YB during spring suggests that healthier *P*. *elliottii* possess superior nutrient uptake efficiency, greater nitrogen demand, and enhanced resilience to climatic extremes.

### 3.6. Network Analysis

**Figure 6:**
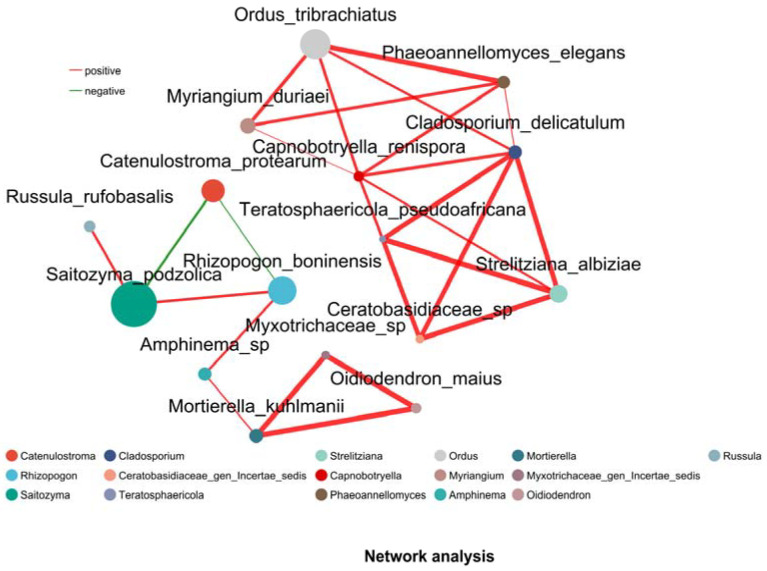
Species-level co-occurrence network: rhizosphere soil communities. Red lines indicate positive correlation, while green lines indicate negative correlation. Thicker lines represent stronger correlations.

Network analysis (Figure 6) revealed 20 significant positive correlations (Pearson, *p* < 0.05) among fungal species across 16 soil samples from YJ and YB. The two species with the highest betweenness centrality were *R*. *boninensis* and *Saitozyma podzolica*, indicating their roles as keystone taxa in the co-occurrence network. Notably, *Saitozyma*, the most abundant genus in our compositional analysis, demonstrated positive facilitative effects on both *R*. *boninensis* and *Russula rufobasalis*.

## 4. Discussion

At the phylum level, significant seasonal variations (*p* < 0.05) were observed between YB and YJ soils, which comprised four major groups: *Ascomycota*, *Basidiomycota*, *Mortierellomycota*, and unclassified taxa. Genus-level analysis revealed pronounced differences (*p* < 0.05) in the relative abundance of *Saitozyma*, *Catenulostroma*, *Neocatenulostroma*, *Rhizopogon*, *Rachicladosporium*, *Tricholoma*, *Archaeorhizomyces*, *Mortierella*, and *Strelitziana*. *Basidiomycota* dominated the spring samples, while *Ascomycota* prevailed in autumn, consistent with Halifu et al.’s findings [49]. When comparing and analyzing the fungal community structure in the rhizosphere soil of two plots at the genus level, it was found that the dominant microbial communities of the two plots showed significant differences. Moreover, the fungal community in the same plot underwent significant changes in different seasons, with all dominant fungal communities being replaced. More *Rachicladosporium* and *Neocatenulostroma* fungi were identified in the YB-10 samples, both of which have been reported to be plant pathogenic fungi causing tree species death [50,51,52]. However, when the abundance of *Rachicladosporium* decreases, plants exhibit higher disease resistance [53]. More *Lophodermium* fungi were identified in YJ-10. Although *Lophodermium* is a pathogenic fungus of various pine trees, studies have shown its potential to induce plant immunity and enhance disease resistance [54,55]. Ectomycorrhizal fungi were identified in both YB-4 and YJ-4 samples, and *Tricholoma*, as an ectomycorrhizal fungus, cannot effectively promote plant growth and nutrient absorption [56]. In addition, *Tricholoma*, as an unconventional ectomycorrhizal fungus, manifests more in parasitic than symbiotic plant relationships. When certain genetic regions are missing, it can also transition from symbiotic to parasitic relationships with plants [57,58]. There is more evidence to suggest that the *Rhizopogon* fungus identified in the YJ-4 sample, also as an ectomycorrhizal fungus, can increase the photosynthesis rate of plants, promote the absorption of nutrients in pine trees, and protect pine seedlings and seeds from the influence of pathogens [59,60,61]. From this perspective, the composition of the fungal community structure in rhizosphere soil is closely related to the health status of *P. elliottii*. From the analysis results, whether in the samples from April or October, the rhizosphere soil of the YJ group contains more fungi that regulate plant immunity and promote nutrient absorption. On the other hand, the dominant fungal community in the rhizosphere soil of the YB group is more harmful or unintentionally harmful to plants. It is worth noting that different types of ECMF were detected in the samples collected from the two plots in April but were replaced by other fungi in the subsequent development process. According to previous investigations and studies, pine blight in China began to occur from March to May, and the incidence rate declined after October [62]. The symbiotic relationship between ECMF and plants can effectively improve their nutrient absorption and utilization, and enhance their stress resistance. From this perspective, appropriate ECMF can effectively enhance the resistance of *P. elliottii* to pine shoot blight pathogens.

Seasonal variations in soil moisture, nitrogen/phosphorus availability, and litter input collectively contribute to ECMF community assembly [63]. The relationship between the dominant fungal genera and environmental factors in two plots was analyzed using RDA. It is interesting that *Rhizopogon* has a higher positive correlation with environmental factor AK, while it has a strong negative correlation with TK and TP. Moreover, *Tricholoma* has a higher positive correlation with the environmental factor AP. Potassium, as an essential plant nutrient, can increase the polyphenol concentration of plants when present in sufficient concentration, playing a key role in defense mechanisms. The extensive use of potassium fertilizer can effectively reduce the occurrence of various diseases [64,65]. The increase in phosphorus concentration will increase the susceptibility of plants [66], and many studies have shown that *Rhizopogon* can promote plant uptake of potassium fertilizer. It seems that *Rhizopogon* plays a crucial role in combating pine shoot blight in pine trees [60,67].

FUNGuild functional prediction identified seven trophic modes, with the pathotroph/saprotroph/symbiotroph guild being predominant, followed by specialized pathotrophs and saprotrophs. The spring samples exhibited higher ECMF colonization rates, coinciding with the “biological synchronization window” for optimal pine/ECMF symbiosis. This phenological pattern provides critical temporal benchmarks for both forest ecology research and mycorrhizal seedling cultivation. Notably, ericoid mycorrhizal abundance in YJ surpassed YB during spring, suggesting these ectomycorrhizal associations not only enhance nutrient acquisition and plant growth but also confer indirect benefits for host disease resistance and environmental stress tolerance.

## 5. Conclusions

Our study explains the differences in fungal community composition in the rhizosphere soil of healthy and susceptible *P. elliottii*, as well as their seasonal variations. At the same time, we demonstrated that there is a correlation between dominant fungal communities and environmental factors. The final results showed that the rhizosphere soil of healthy *P. elliottii* contains more fungal communities that enhance plant disease resistance. The increase in *Rhizopogon* abundance is accompanied by an increase in AK concentration and a decrease in TP content in the soil environment. The changes in soil nutrients directly affect the disease resistance of plants, which may explain why some *P. elliottii* in the same area maintain their health. An incorrect fungal community structure can cause mismatches in soil environmental factors, thereby affecting the disease resistance of *P*. *elliottii*. The research results can provide theoretical guidance for the construction of soil fungal communities in *P*. *elliottii* planting areas and the sustainable management of pine shoot blight in the future.

## Figures and Tables

**Table 1:** Physicochemical properties of susceptible and healthy *P*. *elliottii* in different seasons.

Sample	pH	SOC (g/kg)	AP (mg/kg)	TP (g/kg)	AK (mg/kg)	TK (g/kg)	AHN (mg/kg)
YJ-4	4.66 ± 0.05 a	18.8 ± 0.85 c	1.38 ± 0.06 c	0.20 ± 0.00 c	80.65 ± 4.50 a	5.48 ± 0.60 d	64.09 ± 2.87 c
YB-4	4.20 ± 0.05 c	36.6 ± 1.75 b	5.61 ± 0.18 a	0.25 ± 0.01 bc	71.42 ± 11.08 ab	9.73 ± 3.17 c	102.47 ± 3.88 a
YJ-10	4.30 ± 0.02 b	14.56 ± 0.33 d	1.74 ± 0.64 c	0.23 ± 0.02 b	55.23 ± 0.93 bc	30.32 ± 0.73 ab	61.56 ± 9.98 c
YB-10	4.05 ± 0.03 d	40.29 ± 0.53 a	2.91 ± 0.64 b	0.34 ± 0.02 a	45.08 ± 1.61 c	33.59 ± 1.99 a	99.52 ± 2.43 ab

YJ represents healthy soil samples and YB represents susceptible soil samples; 4 and 10 indicate spring and autumn, respectively. The data in the table are expressed as mean ± standard deviation. The same applies to the following tables. One-way ANOVA was used to analyze differences between the treatments, and Tukey’s test was used to separate the means. Different letters within columns indicate statistically significant differences (*p* < 0.05). A vertical analysis of the data was performed.

**Table 2:** Diversity index analysis of YB and YJ soil samples.

Sample	Shannon Index	Simpson Index	Chao Index	Coverage Index
YJ-4	3.90 ± 0.33 a	0.08 ± 0.03 bc	514.10 ± 87.62 b	99.98 ± 0.03 a
YB-4	3.84 ± 0.43 ab	0.08 ± 0.04 c	616.66 ± 11.97 a	99.81 ± 0.04 b
YJ-10	2.99 ± 0.67 bc	0.12 ± 0.10 ab	109.28 ± 82.37 c	99.98 ± 0.03 a
YB-10	2.47 ± 0.33 c	0.18 ± 0.08 a	89.74 ± 83.43 c	99.98 ± 0.03 a

YJ represents healthy soil samples and YB represents susceptible soil samples; 4 and 10 indicate spring and autumn, respectively. The data in the table are expressed as mean ± standard deviation. One-way ANOVA was used to analyze differences between the treatments, and Tukey’s test was used to separate the means. Different letters within columns indicate statistically significant differences (*p* < 0.05). A vertical analysis of the data was performed.

## Data Availability

The original contributions presented in this study are included in the article/Supplementary Materials. Further inquiries can be directed to the corresponding author.

## Supplementary Materials

The following supporting information can be downloaded at: https://www.mdpi.com/article/10.3390/microorganisms13112476/s1, Table S1: Classification of Rhizosphere Fungi.
